# Salvage (Total) Laryngectomy Rates Following Organ Preservation Strategies in India: Do We Have the Answer?

**DOI:** 10.1007/s13193-025-02360-2

**Published:** 2025-06-03

**Authors:** Shivakumar Thiagarajan, Rukmini Prabhu

**Affiliations:** Division of Head & Neck, Department of Surgical Oncology, Homi Bhabha National Institute (HBNI), Tata Memorial Centre, Mumbai, India

**Keywords:** Organ preservation, Salvage laryngectomy, Stage III/IV, Cancer, India

## Abstract

Laryngeal and hypopharyngeal cancers are among the top 20 cancers in India. Most of these patients present with stage III/IV cancer. Many among these patients may be suitable for the organ preservation protocol. There is sparse data from India on the regimen used for organ preservation strategies, and the salvage of the larynx for any failures after this. In this study, we attempted to identify the regimens used for organ preservation for laryngeal and hypopharyngeal cancer, the larynx preservation rates and the salvage laryngectomy rates for local/locoregional failures. We initially performed a literature search to identify what is available, followed by a survey to understand the present practice patterns and the results. The published literature showed that weekly cisplatin is being utilised for organ preservation strategies commonly, in contrast to the once in 3-week 100 mg/m^2^ that were used in the landmark trials. The salvage laryngectomy rates reported in the literature ranged between 1.8 and 54%. Also, the survey had similar findings. The regimen used for organ preservation was not the one used in the landmark trials. The literature is limited concerning the reporting of the salvage laryngectomy rates following organ preservation strategies.

**Supplementary Information:**

The online version contains supplementary material available at 10.1007/s13193-025-02360-2.

## Introduction

Randomised controlled trials on larynx (organ) preservation in locally advanced laryngeal and hypopharyngeal cancer in the 1990 s and early 2000 s have brought about a paradigm shift in their management from total laryngectomy to concurrent chemoradiotherapy as the standard of care. The Veterans Affairs (VA) trial paved the way for the organ preservation strategy as a treatment option for laryngeal cancer [1]. Subsequently, the EORTC 24981 trial was published [2]. Both trials showed the feasibility of organ (larynx) preservation without compromising survival. Later, the RTOG 91–11 trial established concurrent chemoradiotherapy as the standard of care for locally advanced laryngeal cancer, with a larynx preservation rate of 88% at 2 years without compromising survival [3] (Table 1). The long-term follow-up of the EORTC-24981 and RTOG 91–11 trials showed similar efficacy for the composite endpoints [4, 5]. After the results of the three landmark trials, organ (larynx) preservation was considered as a treatment option for locally advanced laryngeal and hypopharyngeal cancer and concurrent chemoradiotherapy (CCRT) with 100 mg/m^2^ cisplatin once in 3 weeks was considered the standard option for this. However, this is associated with significant acute toxicity in a large majority of the patients, hence giving rise to a suboptimal cumulative dose of cisplatin and thus compromising outcomes [6]. In all these organ (larynx) preservation trials, there was a significant percentage of patients who had to undergo salvage laryngectomy for locoregional failure (Table 1).

**Table 1:** Summary of the three important randomised controlled trials on organ (Larynx) preservation

Organ preservation trials	Treatment arms	Site	Results	Salvage laryngectomy rates
VA trial	NACT* → PR/CR → RT (*n* = 166) → < PR → TL (*n* = 166)	Glottis Supraglottis	2-year OS 68% Both arms 64% larynx preservation	Stage IV: 44% Stage III: 29%
EORTC trial	NACT → CR → RT (*n* = 100) → < PR → TL (*n* = 94)	Supraglottis pyriform sinus	5-year OS in NACT arm 38%, surgery arm 32.6% 48% larynx preservation	13.3%
RTOG trial	NACT → PR/CR → RT (*n* = 173) CTRT^#^ (*n* = 172) RT (*n* = 173)	Glottis Supraglottis	5-year OS: 55% for NACT → RT, 54% for CCRT and 56% for RT 2-year larynx preservation for NACT → RT 75% CCRT 88% and RT 70%	NACT → RT 25% CCRT 16% RT 31%

*NACT* neoadjuvant chemotherapy, *2–3 cycles of cisplatin and 5-fluorouracil, *PR* partial response, *CR* complete response, *RT* radiotherapy, *CCRT* concurrent chemoradiotherapy, ^#^100 mg/m^2^ once in 3 weeks, *OS* overall survival

In India, laryngeal and hypopharyngeal cancers are the 13 th and 15 th most common cancers, and they usually present with stage III or IV disease [7, 8]. Many of these patients present with bulky/large nodal burden, which was not adequately represented in the landmark trials. Also, most patients in the real world may not be fit and tolerate the once-in-3-week 100 mg/m^2^ cisplatin intensive chemotherapy regimens as reported previously. There is also a lack of proper infrastructure and expertise to offer such treatment regimens [9]. Because of this, clinicians prefer once a week 30–40 mg/m^2^ for concurrent chemoradiotherapy [10]. However, the larynx preservation rates and local or locoregional failure and the salvage rates (salvage laryngectomy) for these failures with the weekly cisplatin regimen are not available in the literature. Hence, in this article, we reviewed the available Indian literature to understand what is the practice regarding organ preservation for locally advanced laryngeal and hypopharyngeal cancers and the reported salvage laryngectomy rates.

## Methods

We initially performed a literature search in PubMed only to identify eligible articles. We included articles published from institutes or centres from India between 2000 to 2025 (till date), which reported on adult patients who received definitive treatment for organ preservation (radiotherapy/concurrent chemoradiotherapy) for locally advanced laryngeal and hypopharyngeal carcinoma and also reported on the salvage laryngectomy rates from India. The larynx preservation rate in these patients was also utilised if reported. This literature search was performed to understand the reported literature on organ preservation for stage III/IV laryngeal and hypopharyngeal cancers, the organ preservation protocol used, larynx preservation rates and the salvage laryngectomy rates for local and/or locoregional failures in adults. For this, we used the following MeSH terminologies [((((((Salvage laryngeal surgeries) OR (Salvage laryngectomy)) AND (Concurrent chemoradiotherapy)) AND (Advanced cancer)) AND (Larynx)) OR (hypopharynx)] AND (India)))))))].

We also sent out a survey with 6 questions related to practice patterns for organ preservation strategies for advanced laryngeal and hypopharyngeal cancer to understand the Indian Scenario (https://forms.gle/fwZXazQa7G5BrQWU8) (Supplementary Material). It was an open survey, not a mandatory one, with an initial section describing the reason for conducting the survey. No personal details of the participants were collected. As the survey had only 7 practical questions and was unlike a questionnaire, the validation process was not done. The survey was sent via email to 46 individuals (surgeons) from academic centres, randomly selected, from across the 5 zones in India, from the list of centres within the National Cancer Centres (NCG). Two reminders were given at 2-week intervals and were closed after 1 month. No advertising of the survey was carried out. The sample was a convenience sample with representation of centres across the various zones within the NCG. The descriptive statistics were used to tabulate the information from this survey; no additional statistical analysis was done.

## Results

### Literature Review (Table 2)

**Table 2:** Summary of studies from India on advanced laryngeal and hypopharyngeal carcinoma on organ preservation protocol

Authors	Type of study	Sample size	Site	CCRT schedule	Organ preservation rates	Salvage laryngectomy %
Bahadur S. et al. 2002 [16]	Retrospective	195	Mostly stage II–IV Hypopharynx	Only RT 60–66 Gy	-	47–52
Bhalavat RL et al. 2003 [17]	Randomised trial prospective	64	Supraglottis pyriform sinus T3/T4	Only RT (arm II) 60–66 Gy	53%	20
Gupta T. et al. 2009 [18]	Retrospective	501	T1–T4 (mostly stage III–IV) Hypopharynx	Mostly only RT	-	12.5
Krishnatreya M. et al. 2018 [19]	Retrospective	462	All stages Hypopharynx	Mostly RT	-	1.8
Nair S. V. et al. 2018 [20]	Retrospective	120	T3 Larynx and hypopharynx	70 Gy, cisplatin 30 mg/m^2^, weekly	57.2%	42
Sinha S. et al. 2020 [21]	Prospective	100	Stage III–IV Larynx and hypopharynx	70 Gy, cisplatin 30 mg/m^2^, weekly	61%	43
Fasuludeen A et al. 2022 [22]	Retrospective	630	Mostly T3 (65.6%) Larynx	60–66 Gy, weekly and 3 weekly	-	30.3
Thiagarajan S. et al. 2022 [23]	Retrospective	120	T4 Larynx and hypopharynx	70 Gy, cisplatin 30 mg/m^2^, weekly	-	8
Laskar S. G. et al. 2022 [24]	Prospective	40	(All stage II–IV) Hypopharynx	66 Gy, weekly 40 Mg/m^2^ cisplatin	65%	7.1
Sood S. et al. 2023 [25]	Retrospective	191	Predominantly stage III–IV Larynx and hypopharynx	70 Gy, cisplatin 30–40 mg/m^2^, weekly	56.2%	41.6

**Fig. 1:**
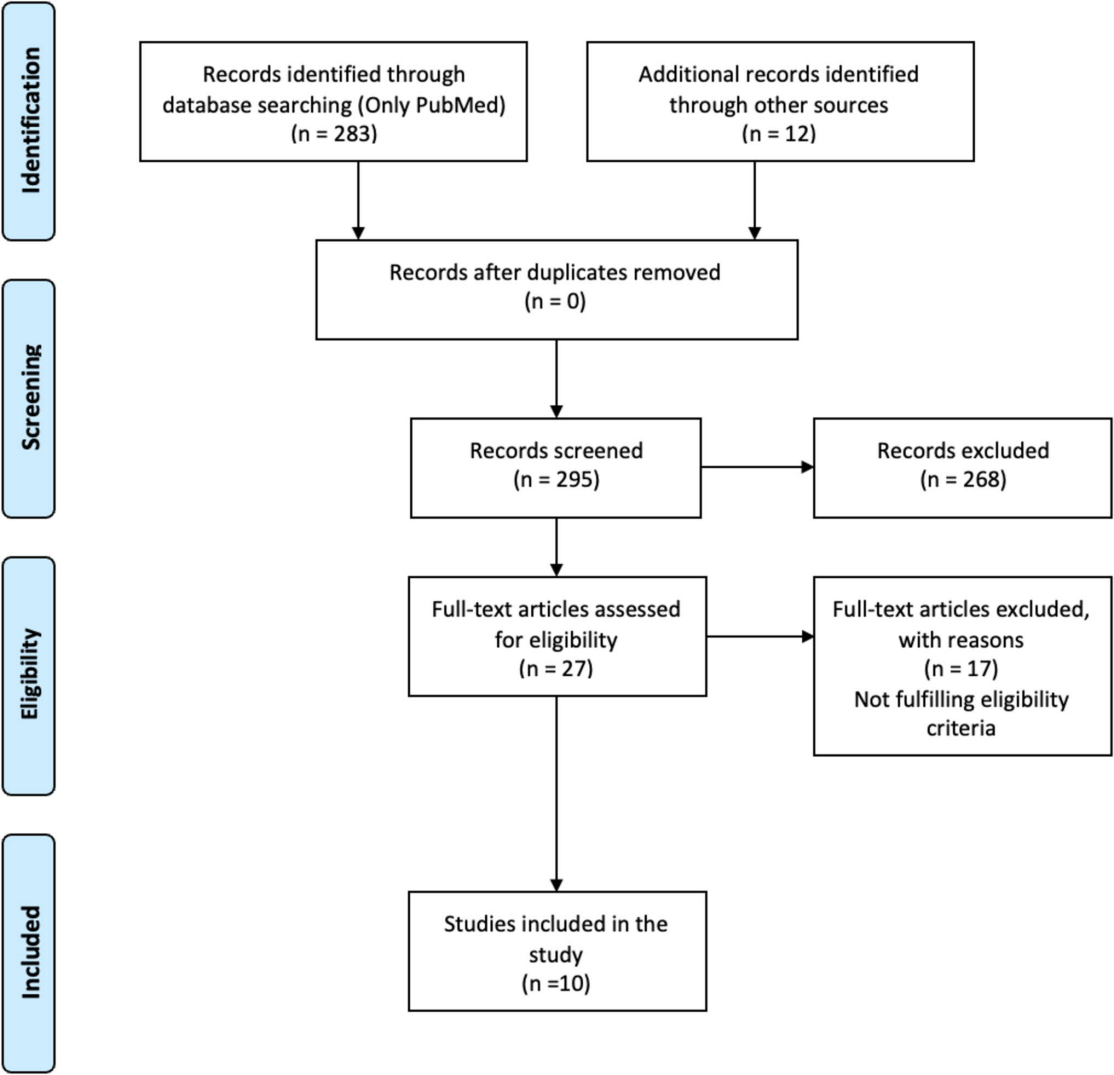
10 articles that satisfied the eligibility criteria

The literature search with MeSH terminologies yielded 283 articles, and an additional 12 articles were identified. After going through the titles and abstracts of the 295 articles, the full text of 27 articles was assessed for eligibility. Finally, 10 articles that satisfied the eligibility criteria were included in the study (Fig. 1). There was one randomised trial, two prospective studies and 7 retrospective studies. The studies published earlier predominantly used only radiotherapy for organ preservation. However, subsequently, chemoradiotherapy was used for organ preservation. However, a point to note here is that weekly cisplatin was the most often used during concurrent chemoradiotherapy. None of the articles reported the median cumulative dose achieved with the weekly cisplatin regimen in their series. Larynx preservation rates were reported between 53 and 65%. The salvage total laryngectomy rates reported in these studies were quite variable. The rates were between 1.8 and 52% in the studies where only radiotherapy was utilised for organ preservation, and it was 7.1–43% where concurrent chemoradiotherapy was utilised.

### Survey (Table 3)

**Table 3:** Summary of the findings of the survey

Questions	Responses
Type of institute Public funded Private NGO/trust	14 (41.2%) 17 (50%) 3 (8.8%)
How many of them would be fit for organ preservation protocol? ≤ 30% 31–60% > 60%	6 (17.6%) 10 (29.4%) 18 (52.9%)
How many of these patients would eventually receive organ preservation protocol? ≤ 30% 31–60% > 60%	4 (11.8%) 8 (23.5%) 22 (64.7%)
Concurrent chemotherapy regimen Weekly Three weekly	27 (79.4%) 4 (11.8%)
Larynx preservation rates (at 3 years) ≤ 30% 31–60% > 60%	1 (2.9%) 17 (50%) 15 (44.1%)
What percentage of recurrence/residual disease would be amenable for salvage laryngectomy? ≤ 30% 31–60% > 60%	16 (47.1%) 14 (41.2%) 4 (11.8%)

We received 34 responses out of 46 (74% response rate) to whom the survey was sent. Among these, 18 (52.9%) of the responders said that > 60% of their patients who attend their institutes/centres would be fit for an organ preservation protocol. The majority were administering weekly cisplatin for the organ preservation strategy at their respective centres, approximately. Most reported organ preservation rates between 30 and 60% at their centres, approximately. With regard to salvage surgery for residual or recurrent disease following organ preservation strategies, 16 (47.1%) of the responders said that < 30% of the patients with recurrence or residual disease would undergo salvage total laryngectomy, and 14 (41.2%) said that 31–60% would undergo salvage surgery. We did not use any cookies, IP checks, or perform log file analysis.

## Discussion

Management of laryngeal and hypopharyngeal cancers has undergone a paradigm shift in the late 1990 s and the turn of the millennium, with a shift in focus from surgery to chemoradiotherapy and preserving the larynx. However, a failure of these strategies mandates salvage surgery. These organ preservation treatment protocols may not be uniformly applicable in developing countries like India, where the presentation of these cancers might be in a more advanced stage. Most centres might also lack the necessary infrastructure to offer such treatment regimens. In a survey-based study that gathered responses from 100 clinicians across India and other developing countries, 91% believed that less than two-thirds of their patients were fit to undergo organ preservation. Fifty percent of clinicians thought that less than 10% of patients with recurrent/residual disease undergo salvage procedures. The common causes for not undergoing salvage surgery were inoperable disease, poor performance status and financial reasons [10].

In our survey, 27 (79.4%) of the clinicians said that their patients received weekly chemotherapy regimens as opposed to three-weekly regimens. In a non-inferiority phase III multicentric randomised control trial of weekly versus three-weekly cisplatin and radical radiotherapy in locally advanced head and neck squamous cell carcinoma, Sharma A. et al. demonstrated that weekly cisplatin is non-inferior to the three-weekly cisplatin regimen, with few hospitalisations, treatment interruptions and toxicity [11]. However, the details of how many patients with advanced laryngeal and hypopharyngeal cancer were included in the study are yet to be seen. Noronha V. et al. in a phase III randomised controlled non-inferiority trial comparing once-a-week versus once-every-3-weeks cisplatin chemoradiation for locally advanced head and neck cancer found that 3 weekly regimens showed better locoregional control (73.1% vs 58.5%). However, 279 (93%) out of the 300 patients received chemoradiation in the adjuvant setting, and so they concluded that the 3-weekly regimen should remain the standard of care in the adjuvant setting [12].

Although there is a wide variation in the treatment protocols, salvage total laryngectomy tends to result in favourable oncologic and functional outcomes compared to other subsites. Patil V. et al. in an analysis of 113 patients with residual or recurrent head and neck cancer have shown that salvage surgery improves overall survival in patients with residual or recurrent disease after CRT. The median OS was improved by > 12 months in patients who were willing to undergo salvage surgery [13]. Goodwin et al. in a meta-analysis demonstrated that the larynx was more amenable to salvage surgery than either the oral cavity or pharynx with 5-year OS rates of 48% in the larynx compared to 43% and 26% in the oral cavity and pharynx subsites [14]. In a retrospective study by Pantvaidya G. et al. of 169 patients who underwent a total laryngectomy, 95 (56.2%) were upfront and 52 (30.7%) were salvage total laryngectomies, highlighting the fact that a good proportion of the patients do undergo salvage laryngectomies [15]. However, the question remains what the overall number of salvage laryngectomies is among patients who receive organ preservation treatment.

There is a dearth of data in the Indian context about salvage laryngectomy rates following concurrent chemoradiotherapy for organ preservation. Table 2 shows the various Indian studies that have looked at organ preservation treatment strategies in laryngeal and hypopharyngeal cancer and their rates of salvage total laryngectomy (1.8–52%) [16–25].

Conservative laryngeal surgeries can also be attempted in selected patients in the salvage setting. Varghese B. T. et al., in their retrospective study, had reported the possibility of conservation laryngeal surgery in the salvage setting. They concluded that conservative laryngeal surgery is oncologically safe and has good functional outcomes. However, they also cautioned that in the salvage setting, patient selection for conservative laryngeal surgery should be done carefully, taking into consideration the disease-free interval, extent of disease, baseline laryngeal function and the pulmonary reserve [26].

This study opens up the issue of limited reporting of the treatment regimens used for organ preservation, organ preservation rate and the incidence or need for salvage total laryngectomy following organ preservation protocol. It also brings to the forefront the need for more transparency in this matter, as it is prudent to know the organ preservation rates achieved with this regimen that has not been tested in the landmark trials.

## Conclusion

The availability of organ preservation data in the public domain of advanced laryngeal and hypopharyngeal cancer in India is sparse. Most centres are using the weekly cisplatin with radiotherapy. Hence, individual institutes must make an effort to report this information more often. Salvage total laryngectomy is the most common salvage surgery done for recurrence/residual disease. The salvage laryngectomy rates reported in Indian literature are variable, 1.8–52%.

## Supplementary Information

Below is the link to the electronic supplementary material.

Supplementary file1 (PDF 55 KB)
