# Supplementary material for: Salvage (Total) Laryngectomy Rates Following Organ Preservation Strategies in India: Do We Have the Answer?
Source: Indian J Surg Oncol. 2025 Jun 3;17(3):599–604. doi: 10.1007/s13193-025-02360-2 (PMC13103054; doi:10.1007/s13193-025-02360-2)
Supplement: Supplementary file 1 — Supplementary file1 (PDF 55 KB) [file 13193_2025_2360_MOESM1_ESM.pdf]

Understanding the Indian Scenario of Larynx & Hypopharynx Organ Preservation.

Dear Sir/Madam,
Greetings! I am trying to understand the organ preservation scenario for the larynx and hypopharynx in India. There are seven questions with free text options to enter the most appropriate answer. This will help understand the organ preservation strategies and the number of patients amenable to salvage subsequently for any recurrences/failures.
Thank you for taking the time to answer these questions.
Regards
Dr Shivakumar Thiagarajan

* Indicates required question

1. Please mention your centre type (Government/Private/NGO/Trust) and Place

2. What is the approximate percentage of patients with advanced (Stage III & IV) carcinoma Larynx & Hypopharynx that you would see in your practice? *

3. What (approximate) percentage of these advanced carcinoma larynx and hypopharynx would be suitable for organ preservation protocol and be offered the same in your practice? *

4. What (approximate) percentage of these patients who are considered suitable and fit for organ preservation end up receiving the same at your institute? *

5. Do you give weekly (40mg/m2) or once every three weeks (100 mg/m2) cisplatin in the organ preservation regimen? *

6. What is the approximate larynx preservation percentage at three years post-treatment completion? *

7. What is the approximate percentage of recurrence/failures following organ preservation, and what percentage of these patients are amenable to salvage surgery subsequently (salvage laryngectomy)? *

This content is neither created nor endorsed by Google.

Google Forms
